# Trophic and tectonic limits to the global increase of marine invertebrate diversity

**DOI:** 10.1038/s41598-017-16257-w

**Published:** 2017-11-21

**Authors:** Pedro Cermeño, Michael J. Benton, Óscar Paz, Christian Vérard

**Affiliations:** 1 Institut de Ciències del Mar, Consejo Superior de Investigaciones Científicas, Passeig Marítim de la Barceloneta 37-49, 08003 Barcelona, Spain; 2 School of Earth Sciences, University of Bristol, Bristol, BS8 1RJ United Kingdom; 3 Institute for Environmental Sciences (ISE), University of Geneva, Boulevard Carl-Vogt, 66, CH–1211 Genève/GE, Switzerland

## Abstract

The marine invertebrate fossil record provides the most comprehensive history of how the diversity of animal life has evolved through time. One of the main features of this record is a modest rise in diversity over nearly a half-billion years. The long-standing view is that ecological interactions such as resource competition and predation set upper limits to global diversity, which, in the absence of external perturbations, is maintained indefinitely at equilibrium. However, the effect of mechanisms associated with the history of the seafloor, and their influence on the creation and destruction of marine benthic habitats, has not been explored. Here we use statistical methods for causal inference to investigate the drivers of marine invertebrate diversity dynamics through the Phanerozoic. We find that diversity dynamics responded to secular variations in marine food supply, substantiating the idea that global species richness is regulated by resource availability. Once diversity was corrected for changes in food resource availability, its dynamics were causally linked to the age of the subducting oceanic crust. We suggest that the time elapsed between the formation (at mid-ocean ridges) and destruction (at subduction zones) of ocean basins influences the diversity dynamics of marine invertebrates and may have contributed to constrain their diversification.

## Introduction

Based primarily on the metazoan fossil record, two models of clade diversification, equilibrium and non-equilibrium, have been proposed to explain the evolution of taxonomic diversity since the Cambrian (~541 million years, Myr, ago)^1–5^. The equilibrium model predicts that, for a given amount of resource availability and ecospace occupation, diversity cannot exceed a global carrying capacity and, as a consequence, diversity eventually reaches an equilibrium level^1,6^. At equilibrium, the origination and settling of new species are balanced by the failure and extinction of earlier taxa in what has been termed an evolutionary arms race. The non-equilibrium model, in contrast, predicts that diversity may rise or fall as abiotic forcing mechanisms such as tectonic, eustatic, climatic and/or oceanographic contingencies promote either lineage splitting or termination, respectively^7–10^. Alternatively, a non-equilibrium, innovation-driven model has been put forward to account for instances of unconstrained diversity growth in the absence of abiotic forcing mechanisms^11^. In all cases, diversity levels are expected to track changes in food resource availability^12–14^, making it difficult to select the model that best explains the diversity dynamics of marine animals. For instance, enhanced food supply relieves biological communities from resource limitation, which is the main control on diversity expansion in equilibrium models^15,16^. Alternatively, enhanced food supply could promote diversification, if tectonic processes facilitated habitat fragmentation^8^ or, alternatively, if evolutionary innovation led to novel mechanisms for resource exploitation^17,18^. Whereas increased resource supply is an essential requirement for the growth of diversity in equilibrium models, resource limitation does not necessarily preclude the growth of diversity in non-equilibrium diversification models. As a consequence, an increase of diversity under conditions of constant resource supply should be indicative of abiotic forcing mechanisms or new ecospace occupation. Here we correct marine invertebrate diversity for changes in marine food supply through Phanerozoic time (~541 Myr ago to present), thereby revealing the signal of diversity dynamics related to factors other than food resource availability. Then, we use a state-of-the-art statistical method for causal inference to unveil the mechanisms responsible for changes in corrected diversity. In particular, we explore the effect of mechanisms associated with the history of the seafloor (i.e. biome age), and their influence on the creation and destruction of marine benthic habitats using data derived from a geodynamic model.

## Results

**Figure 1:**
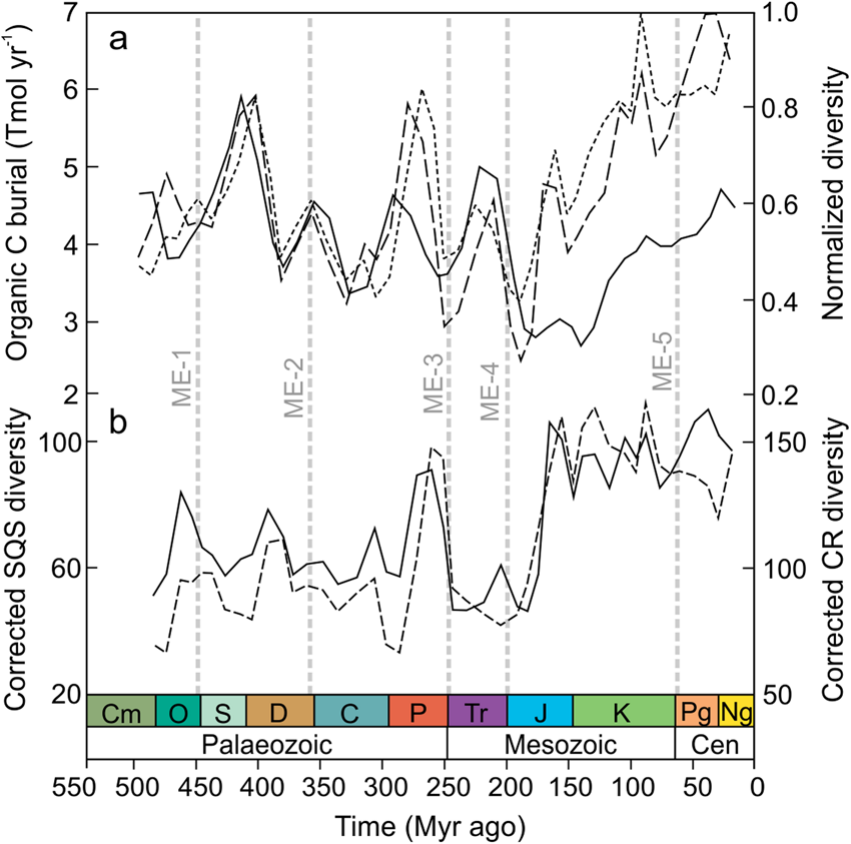
Phanerozoic trends in marine organic C burial rate and the global diversity of marine invertebrates. (**a**) Organic C burial rate after subtracting the fraction of organic C buried in non-marine environments (solid line) (see Methods). Marine invertebrate diversity estimates based on the shareholder quorum subsampling method (SQS) and the classical rarefaction (CR) (dashed and dotted line, respectively). For representation purposes, diversity estimates were normalized to the maximum value of the series. (**b)** Diversity corrected for changes in marine organic C burial rate through time for SQS and CR diversity estimates (solid and dashed line, respectively). ME1-5 denotes the temporal distribution of mass extinction events.

Global estimates of the organic carbon (C) burial rate, extracted from the long-term C and sulphur (S) cycle GEOCARBSULF model^19^, were used as a proxy for food supply. Not all sources of detrital organic matter were accessible to marine consumers. C and S isotope mass balance analyses show a maximum of O_2_ centred around 300 Myr ago, which was caused by the expansion of terrestrial vascular plants^20^ combined with a combination of climatic and palaeogeographic factors that enhanced plant-derived organic C preservation and coal formation^21^. It is possible to estimate the rate of organic C burial in marine environments through time using the worldwide weight ratio of organic C to pyrite-S (C/S) in sedimentary organic matter (see Methods). Whereas abundant sulphate reduction and pyrite formation in marine euxinic environments leads to sedimentary organic matter with low C/S ratios^22^, the burial of organic C in freshwater environments such as forest swamps and peatlands results in high C/S ratios because of their comparatively lower sulphate content^22^. The resulting marine organic C burial rate curve showed a cyclical pattern during the Palaeozoic (541–252 Myr ago) and Early Mesozoic (252–175 Myr ago) superimposed on a long-term decline, followed by a secular increase across the Mid-Late Mesozoic (175–66 Myr ago) and Cenozoic (66 Myr ago-present) (Fig. 1a). We compared estimates of organic C burial rate against sampling-standardized estimates of marine invertebrate diversity based on the shareholder quorum subsampling method, SQS, and classical rarefaction, CR. Sampling standardization is recommended because sampling effort per time interval in fossil databases is skewed toward recent records^23^. The organic C burial rate curve was remarkably coincident with the diversity dynamics of marine invertebrates (Fig. 1a), substantiating the idea that global species richness is largely regulated by resource limitation^15,16^. The disparity of the sources from which diversity and organic C burial rate estimates were obtained emphasizes the significance of such a remarkable correspondence.

**Figure 2:**
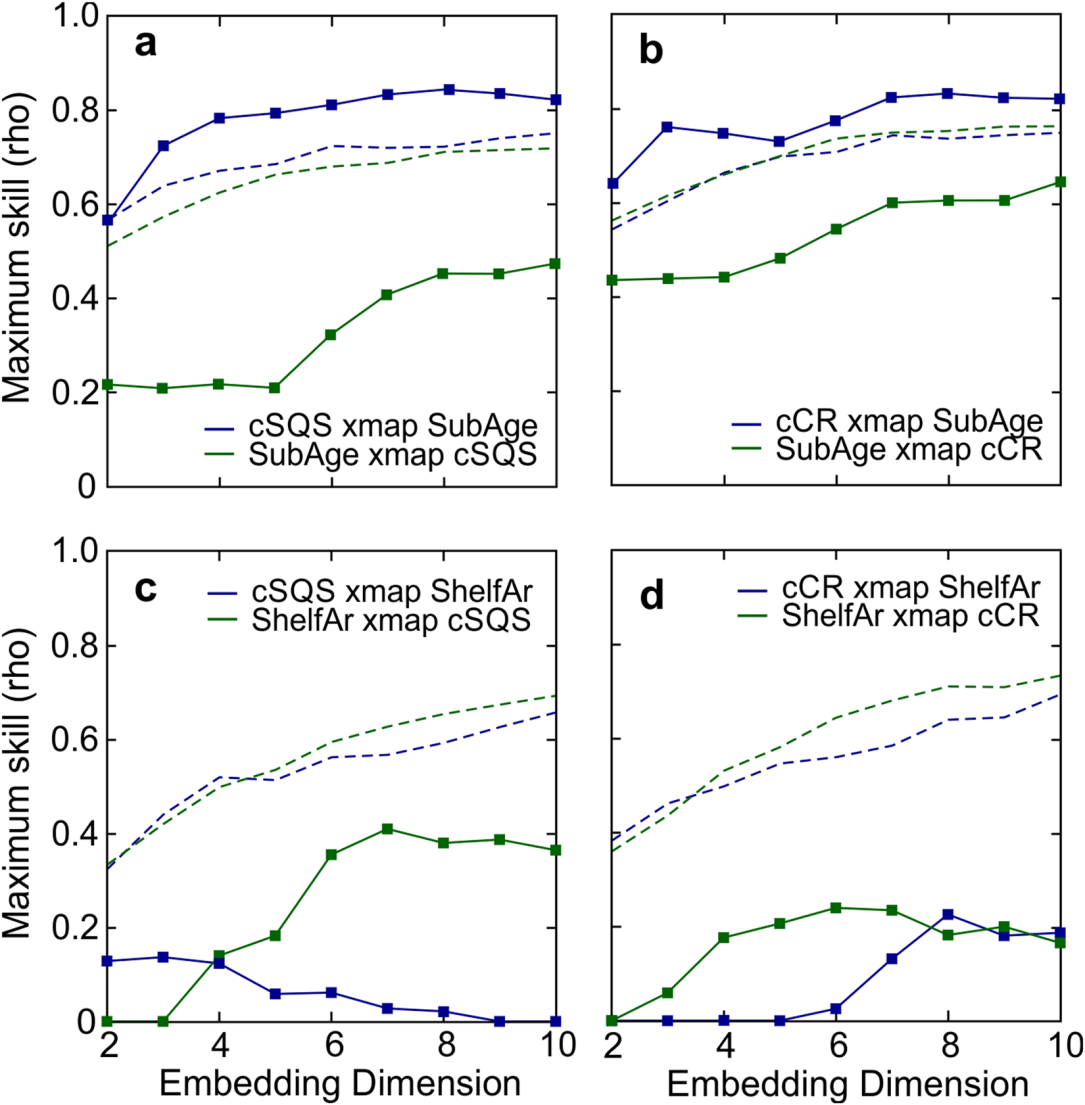
Convergent cross mapping for detecting causality. Maximum cross-mapping skill at different embedding dimensions for the relationships between (**a**,**b)** mean age of the subducting crust (SubAge) and corrected diversity (cSQS and cCR), and (**c**,**d**) global shelf area (ShelfAr) and corrected diversity (cSQS and cCR). The 95th percentile of the corresponding cross mapping skill for 1000 surrogate time series from the Ebisuzaki phase shift null model^71^ is also shown (dashed lines). Cross-mapping skill and causality were considered significant if the Sugihara’s correlation coefficient (rho) for the cross-mapping of time series *X* to *Y* exceeded the 95^th^ percentile of the corresponding estimate for the surrogates (e.g., *X* xmap *Y* means that *Y* causes *X*) (see Methods).

We corrected diversity curves for changes in marine food supply through time as inferred from a resource-associated proxy variable such as the marine organic C burial rate (i.e. sampling-standardized diversity in a given time bin divided by the rate of marine organic C burial in the same bin) (Fig. 1b). The resultant variable represents an estimate of the number of lineages supported by a given rate of marine food supply and underlines the signal of diversity dynamics related to factors other than food resource availability (i.e. equivalent to the hypothetical scenario of constant food supply through time). We first observed that the corrected diversity curves still showed a pattern of peaks and troughs across the entire Phanerozoic superimposed on a long-term secular trend (Fig. 1b). To investigate the cause/s for the variability of corrected diversity, we tested for causality using convergent cross mapping (CCM), a statistical test for cause-and-effect relationships based on the dynamical systems theory and the concept of state space reconstruction^24^. If variable *X* causes *Y*, then the values of *X* can be reconstructed from the state information of *Y* via cross mapping based on a training dataset recorded in the corresponding shadow manifold, but not vice versa (i.e. *Y* xmap *X* ≠ *X* xmap *Y*) (see Methods). CCM analyses revealed significant causal relationships between estimates of corrected diversity and the mean age of the subducting oceanic crust (Fig. 2a,b). These results were robust to changes in embedding dimension, a critical parameter in cross mapping methodology (Fig. 2a,b and Supplementary Fig. S1). However, our analysis showed that neither changes in global shelf area nor sea level height were primary determinants of corrected diversity through time (Fig. 2c,d and Supplementary Fig. S2).

**Figure 3:**
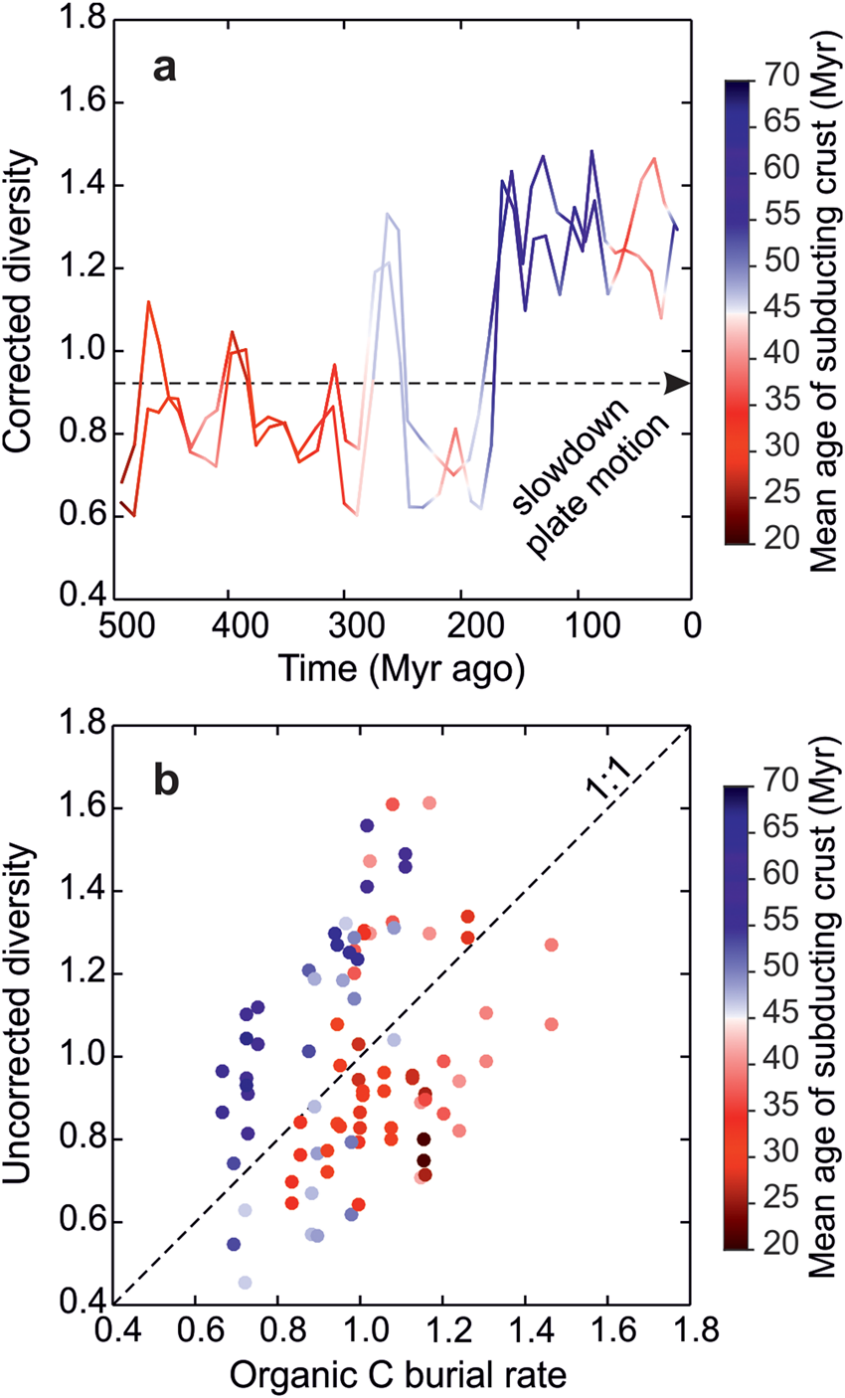
A tectonically-driven model of marine invertebrate diversification. (**a)** Corrected diversity (cSQS and cCR) through the Phanerozoic with colour code depicting the mean age of the subducting oceanic crust. Corrected diversity estimates were normalized to the mean value of each time series. (**b)** Uncorrected diversity estimates (SQS and CR) plotted against marine organic C burial rate. Diversity and organic C burial rate estimates were normalized to the mean value of each time series. Data points above the 1:1 line indicate that global diversity becomes dominated by lineages with a smaller per capita share of food resources relative to the normalization factor, and vice versa for data points falling below the 1:1 line.

The age of the subducting oceanic crust is adjusted by the rates of seafloor spreading and subduction, and can be used as a proxy for the longevity of ocean basins. For instance, low rates of seafloor spreading and subduction increase the time elapsed between the formation and destruction of ocean basins, thereby allowing more time for the growth of diversity. Conversely, high rates of crustal recycling shorten the history of the seafloor and hence the time available for the development of diversity. On average, corrected diversity and the age of the subducting oceanic crust increased towards the present (Fig. 3a), presumably associated with an overall reduction in the speed of plate motions^25,26^.

Finally, for a given amount of food, an increase of uncorrected diversity would imply a finer partitioning of food resources among taxa. Simply, there is the same amount of food for a larger number of taxa. We compared estimates of uncorrected diversity and marine organic C burial rate in order to investigate the extent to which the per-capita share of food resources varied through time (Fig. 3b). In this plot, data points above the 1:1 linear relationship indicate that global diversity became dominated by lineages with a smaller per capita share relative to the normalization factor (i.e. the mean value of the time series), and vice versa for data points falling below the 1:1 line. Our analysis indicates that, on average, modern faunas exhibit lower levels of per-capita share than their Palaeozoic counterparts (Fig. 3b), largely reflecting an increased degree of faunal provinciality.

## Discussion

We have reported a statistically significant positive relationship between the rate of organic C burial and diversity using model estimates of organic C burial rate and marine invertebrate fossil data spanning the entire Phanerozoic eon (Fig. 1a, 3b). To the extent that organic C burial rate represents a proxy for food resource availability, our results substantiate the idea that resource limitation imposes fundamental limits to the global increase of marine invertebrate diversity^1,6,15,16^. Once diversity was corrected for changes in organic C burial rate, we found a strong causal linkage between diversity and the mean age of the subducting oceanic crust (Fig. 2a,b), suggesting a role for plate tectonics in regulating evolutionary rates.

Sedimentary data and satellite-derived estimates of ocean net primary production suggest that the vast majority of organic burial takes place on and immediately adjacent to continental shelves^27,28^. It has been estimated that, at present, 0.13–0.16 Pg C y^−1^ are buried in the oceans, with 80–85% of this occurring along continental shelves and deltas^29^. Likewise, the shallow continental shelf is where the vast majority of marine diversity and productivity of benthos is located^30,31^. Thus, we speculate that variations in organic C burial rate through time primarily influenced the biodiversity of highly productive continental margins, which represent the locus of major organic C burial. This observation does not preclude a role for food resource limitation in regulating the diversity of deep sea benthic communities. Rather, owing to the more homogeneous delivery of organic matter into the deep sea, we suggest a comparatively minor role for resource fluctuations in regulating the evolutionary rates and diversity dynamics of deep sea faunas.

The age of the subducting oceanic crust was computed from global palaeogeographic reconstructions generated by the University of Lausanne (UNIL) geodynamic model (v.2011, © Neftex) (Fig. 2a,b). The UNIL model predicts a long-term reduction in the global average rate of seafloor spreading and subduction, and the ensuing progressive aging of the oceanic basins over the last 600 Myr^26,32^. On the other hand, the observation of a linear decrease in the area of preserved oceanic lithosphere per unit time with increasing age has led to the hypothesis of a constant destruction (and therefore production) rate of the oceanic crust through time^33^. The extent to which this relative stability of tectonic plate motion is extensible to the Palaeozoic era is not straightforward. Spreading rates calculated thanks to preserved magnetic anomalies have varied strongly since Jurassic times^34,35^, and model simulations indicate that the wavelength of predicted variations is much longer than 80 Myr^26^. For the Cretaceous, the results of the UNIL model are in good agreement with previous studies on accretion^36,37^ and subduction rates^38^. Indeed, comparing average plate velocities stemming from the UNIL model to seafloor spreading rates resulting from other geodynamic models for Palaeozoic and Mesozoic times^25,39,40^, we found consistently a long-term decrease in the rates of seafloor spreading and subduction through the Palaeozoic and Mesozoic (Supplementary Fig. S3).

The role of plate tectonics and Earth system evolution in regulating the diversity dynamics of marine animals has long been associated with the demarcation of geographic provinces (e.g. assembly-disassembly of continental masses)^8,41^, tectonic enhancement of nutrient weathering fluxes^42^, the impact of continental configuration on global climate^43^, and changes in sea level^9^, shelf redox conditions^9^ or volcanism^44–46^. Our analysis suggests that the slowdown of plate tectonics further increased the global diversity of marine invertebrates. We interpret this causal connection between plate kinematics and the diversity dynamics of marine invertebrates as a result of the positive relationship between seafloor age and number of geographical barriers to dispersal, which represents a major control on marine animal diversification^47,48^. Separate analyses on specific taxonomic groups (bivalves, gastropods, etc) might potentially unveil additional controls on diversity^49,50^.

In summary, our analysis suggests that rates of seafloor spreading and subduction influenced the diversity dynamics of marine invertebrates throughout the Phanerozoic. According to these results, a tectonically-driven non-equilibrium model of marine invertebrate diversification is proposed. In our model, diversity increases in response to the intensification of continental weathering and the slowdown of plate tectonics, which increase, respectively, the productivity and age of oceanic basins. This route of diversity expansion entails an increase in the number of geographical discontinuities or provinciality, an increase in geographic disparity; an index of the compositional similarity among the biotas of a given stratigraphic interval with respect to their geographic distances from one another^51,52^ and an increase in the time lag between lineage splitting and their subsequent expansion^53^. Conversely, intervals of diversity loss most likely reflect phases of food shortage and rapid crustal recycling. Our results suggest that the relative stability of marine invertebrate diversity through the Phanerozoic might not be a result, at least exclusively, of ecological constraints on diversity growth^1,3,5,6,16^, but a consequence of the continuous recycling and deformation of the oceanic crust, which sets another fundamental limit to the global increase of marine animal diversity.

## Methods

### Data

The time series of sampling-standardized marine invertebrate diversity^1,50^, organic carbon (C) burial rates^19,54^, sea level^55,56^, mean age of the subducting oceanic crust^26^ and global shelf area^57^ through the Phanerozoic were extracted from previous reports by graph digitization using GetData Graph Digitizer v2.26. Additionally, estimates of organic C burial rate in marine environments and corrected diversity were derived from digitized data. The following sections describe the methods used in the papers depicting the graphs digitized here.

#### Diversity data

Sampling effort per time interval in fossil databases is skewed toward recent records. To correct for differences in sampling effort across the time series, sampling-standardized estimates of the number of taxa are commonly used^23^. The classical rarefaction (CR) method uses randomised subsampling protocols that seek to hold each time interval to a uniform sample quota or number of taxonomic occurrences^23,58^. This method, and in general those based on fixed quotas, effectively sample only those taxa with a relatively high frequency (i.e. the bulk of rare taxa are more difficult to detect and frequently missed in datasets). The shareholder quorum subsampling (SQS) method represents an advance in this regard over quota methods. This method calculates the expected number of taxa by sampling a given, fixed coverage of the frequency curve of species occurrences, where coverage is the sum of the frequencies of the species sampled^50,59^. The SQS method produces relative diversity estimates with fair but uneven sampling such that taxa with relatively low frequencies are equally well represented in estimates as dominant ones. Typically, diversity estimates are tabulated from large numbers of pseudorandom datasets generated under the subsampling routines described above (i.e. item quota and fixed coverage, respectively). For representation purposes, CR and SQS diversity estimates were normalized to the maximum (Fig. 1a) or mean value (Fig. 3) of the time series.

#### Organic C burial rate

Global organic C burial rates are calculated by models such as GEOCARBSULF, a long-term C and sulphur (S) cycle model^19,54^, which estimates atmospheric carbon dioxide (CO_2_) and oxygen (O_2_) levels by reconstructing long-term sources and sinks through time. GEOCARBSULF tracks the multi-million-year transfer of C and S between surface reservoirs (atmosphere, ocean, soil, living biomass) and rock reservoirs, mainly organic C, carbonate C and pyrite S. As for C, volcanic degassing constitutes the primary input of C to the surface reservoirs, whereas silicate weathering and subsequent biogenic sedimentation as carbonate C and organic C dominates removal^60–62^. S is released from geological sources through the weathering of continental rocks, volcanic degassing and hydrothermal emanation of S-bearing gases and fluids. Once S is exposed to subaerial conditions, it combines with O_2_ to form sulphate. Plants and microbes assimilate sulphate and convert it into organic compounds. The burial of anhydrite (calcium sulphate, CaSO_4_) and pyrite (iron sulphide, FeS) in the oceanic crust exceeds its removal. Organic C and pyrite-S are isotopically lighter than the CO_2_ and sulphate they are derived from due to biological fractionation during photosynthesis and sulphate reduction. Because the timescale of integration is sufficiently long (typically >10 Myr), the model presumes that the surface reservoirs are in a “quasi” steady-state (i.e. input fluxes of C and S to the surface reservoirs must be balanced in mass and isotopic value by output fluxes)^63^. Hence, the model calculates organic C, carbonate C and pyrite-S burial rates through time by isotope mass balance, a technique in which burial rates are inferred in order to match known isotope records.

The relative contribution of marine and non-marine organic C burial to the sedimentary organic C reservoir is critical to quantify the amount of food resources available for marine invertebrates. It is possible to distinguish the relative contribution of marine and freshwater environments to organic C burial by looking at the patterns of sedimentary pyrite formation through time^27,64^. The rationale is founded on the observation of an inverse relationship between δ^13^C and δ^34^S, which results from a positive covariance of organic C and pyrite-S contents in marine sediments^22^. The main reason of this relationship lies in the fact that a fraction of marine organic matter is mineralised by sulphate-reducing bacteria in marine environments (primarily euxinic). Conversely, enhanced deposition of organic material in freshwater environments (e.g. forest swamps) results in a negative covariance of organic C and pyrite-S because of the relatively lower sulphate content of freshwater systems, where pyrite formation is comparatively negligible. We used the worldwide burial ratio of organic C to pyrite S (C/S) in sediments^65,66^ as a proxy for changes in the dominant locus of organic C deposition through time. Whereas abundant sulphate reduction and pyrite formation in Early Palaeozoic marine environments gave rise to sedimentary organic matter with low C/S values^22^, i.e. prior to the expansion of terrestrial plants, present-day values are the result of both terrestrial and marine organic C sources^22^. Thus, we calculated the rate of marine organic C burial through time as a function of C/S by assuming that min(max) C/S values correspond to min(max) terrestrial organic C burial rates. Then, the terrestrial organic C burial rate curve through time was calculated accordingly. This method is analogous to those based on C and S isotope mass balance analyses^64,67^, which calculate marine organic C burial rate as a function of pyrite burial.

#### Geodynamic data

The mean age of the subducting oceanic crust was calculated by using a global geodynamic model following true plate tectonic principles and covering the whole surface of the Earth (i.e. continental and oceanic realms)^26^. The model was constructed by assembling regional-scale tectonic reconstructions developed at the University of Lausanne (UNIL) over the past 20 years^26,32^ (v.2011, © Neftex). These reconstructions were generated by the analysis of elementary units, also named geodynamic units (GDUs). A GDU describes the present-day smallest continental/oceanic fragment that underwent the same geodynamic evolution through time. The Earth’s history is reconstructed by redistributing the GDUs through space and time. The GDUs position is controlled by palaeogeographic data and geological data of geodynamic interest such as rift zone, passive margin, active margin, collision zone, etc^32^. Knowing the positions of the GDUs and the timing of major geodynamic events (e.g. continental break-up, collision, subduction initiation or reversal, etc.*)*, one can trace the motion of plates. For disappeared oceans, the exact shape of mid-oceanic ridges is not known. However, during continental break-up, if plate velocities and geometries are known, the space between the detaching and the left-over fragments (into which the new ridge formed) can be constrained. The reconstruction work is carried out from 600 Myr ago to present. Crustal material is added/removed in divergent/convergent areas marked by newly defined plate boundaries. From one reconstruction to the next, plate positions are interpolated and former plate polygons are first preserved to identify the diverging (gaps) and converging (overlaps) areas. The UNIL model comprises 48 reconstructions extending back to 600 Myr ago every 5–20 Myr ago. Using a specific software, these global age map reconstructions were then used to compute the mean age of the oceanic crust along subduction zones^26^.

#### Data processing

The time series were arranged into continuous curves using both linear and cubic splines interpolation methods. The former assumes a linear point-to-point connection through the series data, while in the latter the interpolated value at a query point is based on cubic interpolation of the values at neighbouring grid points in each respective dimension^68^. Among all the time series extracted, the time series of diversity (CR and SQS) were the most limited in length. Since CR and SQS are central variables in our study, we considered their sampled time points as our base time array. Consequently, we projected (downsampled) all other time series onto that target time vector using the aforementioned interpolation methods. Subsequent analyses revealed that the interpolation of raw data did not play any significant role so as to modify quantitatively our findings, and therefore we chose the simpler piecewise linear interpolation method as our data estimator throughout the study (Supplementary Dataset).

### Detecting causality between time series: Convergent Cross Mapping

The interplay between key variables was further analysed by considering potential cause and effect combinations. Causal interactions cannot be directly assessed from plain correlations (even in its first differences form) and are not straightforward to evaluate in general. Sugihara and co-workers^24^ proposed the Convergent Cross Mapping (CCM) technique, which relies on central statements in the theory of nonlinear dynamical systems; namely, the delay embedding theorem (also known as Takens’ theorem) and the concept of state space reconstruction. By means of the historical observations of two given coupled variables (*X* and *Y*) evolving in time (*t*), one can construct for a dynamical system the arrays *M*_*X*_ = {*X*(*t*), *X*(*t*-*τ*}, *X*(*t*-2*τ*}, …, *X*(*t*-*Eτ*)} and equivalently *M*_*Y*_ (for the time series *Y*), where *E* is an integer denoted as the embedding dimension and *τ* is the time delay. *M*_*X*_ and *M*_*Y*_ conform lagged coordinate embeddings of *X* and *Y*, respectively. The theorem dictates generically that these vectors, considered as new coordinates in a (*E* + 1)-dimensional space, can in fact be used to reconstruct shadow versions of the original system’s manifold *M* or state space, from which *X* and *Y* series are nearly just time projections of the manifold dynamics onto corresponding coordinate axes. Moreover, and crucially, the scheme of a state space reconstruction ensures that each *M*_*X*_ and *M*_*Y*_ manifolds are in one-to-one mapping to the state of the original dynamical system, which “lives” in the manifold *M* (state space) expanded by *X*(*t*), *Y*(*t*) and eventually other variables *Z*_*i*_(*t*), while also preserving the topology and other essential mathematical properties. The reconstructed shadow manifolds *M*_*X*_ and *M*_*Y*_, each produced from lags of a single variable, can be therefore used to recover states of the original dynamic system. If *X* and *Y*, belonging to the same dynamic system, are two variables causally related, so are thus their shadow manifolds *M*_*X*_ and *M*_*Y*_, since they are in a one-to-one correspondence to each other (through *M*). CCM exploits the fact that points that are nearby on the manifold *M*_*X*_ correspond to points that are also nearby on *M*_*Y*_, as a necessary condition for causality between *X* and *Y*. By finding nearest neighbour points in *M*_*X*_, say coordinates *P*_*X*_, and the corresponding points by same time indexing in *M*_*Y*_, from which to estimate a new set ${P}_{{X|M}_{Y}}$ using simplex projection^69^, one can tell about causation if the set ${P}_{{X|M}_{Y}}$ actually resembles the real nearest neighbours *P*_*X*_ in *M*_*X*_ (just by computing standard Pearson correlation coefficients between both sets). The technique is therefore called cross mapping and is denoted in this case as “*Y* xmap *X*”, meaning that “*X* is causally influencing *Y*”.

The CCM analyses were performed using the rEDM package (https://cran.r-project.org/package=rEDM) written in R programming language. Because of the short-length time series, the delay time lag *τ* was always set to 1 time step (~11 Myr). In order to determine the best embedding dimension *E*, false nearest neighbour tests with parameters of *R*_*tol*_ and *A*_*tol*_ of 15 and 2 respectively^70^ were performed on each time series independently. An optimum overall embedding dimension of *E* = 3 was obtained from these tests for most of the cases involved, although other embedding dimensions were examined as an extra check on parameters dependency (Fig. 2 and Supplementary Fig. S1). At each library size (ranging up to such 45 points), leave-one-out cross validation constructed from 200 different randomly-chosen library samples was employed to avoid any bias in the calculations. The averaged cross map skill at each library size was plotted as the result (Supplementary Fig. S1). For the sake of simplicity, the maximum skill is plotted in Fig. 2. Causal tests for the whole set of variables investigated were carried out at three different embedding dimensions (Supplementary Fig. S2).

Statistical significance of the results was verified by surrogates data testing using the Ebisuzaki phase shift null model^71^. A null distribution of 1000 generated surrogate time series was set to perform this check. Outcomes from CCM calculations were then considered significant if the Sugihara’s correlation coefficient (rho) for the cross-mapping of time series *X* to *Y* exceeded the 95^th^ percentile of the corresponding estimates for the surrogates.

### Uncertainty in organic C burial rate estimates

Global organic C (and reduced S) burial rates can be estimated from models computing the input of nutrients via weathering fluxes (e.g. COPSE model)^72^ or, alternatively, by comparing geological C and S isotope records to the isotopic composition of modelled sediments assuming steady-state (isotope mass balance)^54^. There is considerable quantitative uncertainty regarding estimates of global (and marine) organic C burial rates during the past 200 Myr^19,54,72,73^. For instance, isotope mass balance analyses predict estimates of atmospheric O_2_ concentration and organic C burial rates well below those predicted by nutrient-weathering models^73^. Taking as valid marine organic C burial rate estimates computed from COPSE (in its original configuration), which does not support for relatively low productivity and organic C burial rates during the past 200 Myr, our analysis suggests a significant causal relationship between the mean age of subducting crust and corrected CR diversity (i.e. cCR(copse) xmap SubAge, Supplementary Fig. S2), but non-significant for corrected SQS diversity.

## Electronic supplementary material

Supplementary Information

Dataset
