# Supplementary material for: Trophic and tectonic limits to the global increase of marine invertebrate diversity
Source: Sci Rep. 2017 Nov 21;7:15969. doi: 10.1038/s41598-017-16257-w (PMC5698323; doi:10.1038/s41598-017-16257-w)
Supplement: Supplementary file 1 — Supplementary Information [file 41598_2017_16257_MOESM1_ESM.doc]

Trophic and tectonic limits to the global increase of marine invertebrate diversity

Pedro Cermeño^1^*, Michael J. Benton^2^, Óscar Paz^1^, Christian Vérard^3^

^1^Institut de Ciències del Mar, Consejo Superior de Investigaciones Científicas, Passeig Marítim de la Barceloneta 37-49, 08003 Barcelona, Spain.

^2^School of Earth Sciences, University of Bristol, Bristol BS8 1RJ, United Kingdom.

^3^Institute for Environmental Sciences (ISE), University of Geneva, Boulevard Carl-Vogt, 66, CH–1211 Genève /GE, Switzerland.

*Corresponding author: pedrocermeno@icm.csic.es

**
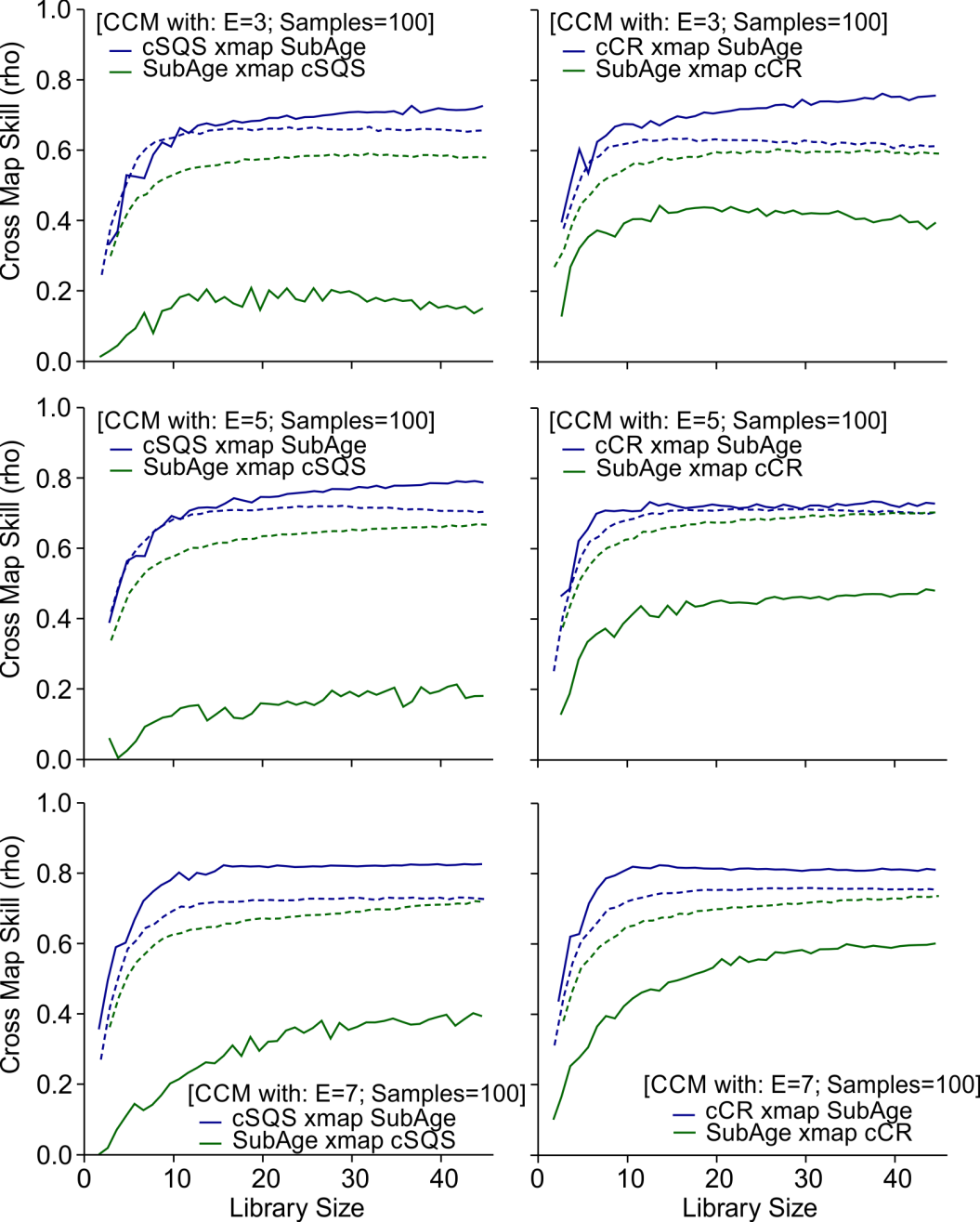
**

**Supplementary Figure S1. Correlation of cross-mapped versus observed values as a function of the length of the time series (namely library size)**. Sugihara’s correlation coefficient (rho) for the cross‑mapping of time series *X* to *Y* (solid lines). The 95th percentile of cross mapping skill for 1000 surrogate time series from the null model is also shown (dashed lines). Causality was considered significant if the Sugihara’s correlation coefficient for the cross‑mapping of time series *X* to *Y* exceeded the 95^th^ percentile of the corresponding estimate for the surrogates (e.g., *X* xmap *Y* means that *Y* causes *X*). Cross mapped variables are corrected diversities (cSQS and cCR), and the mean age of subducting crust (SubAge).

**
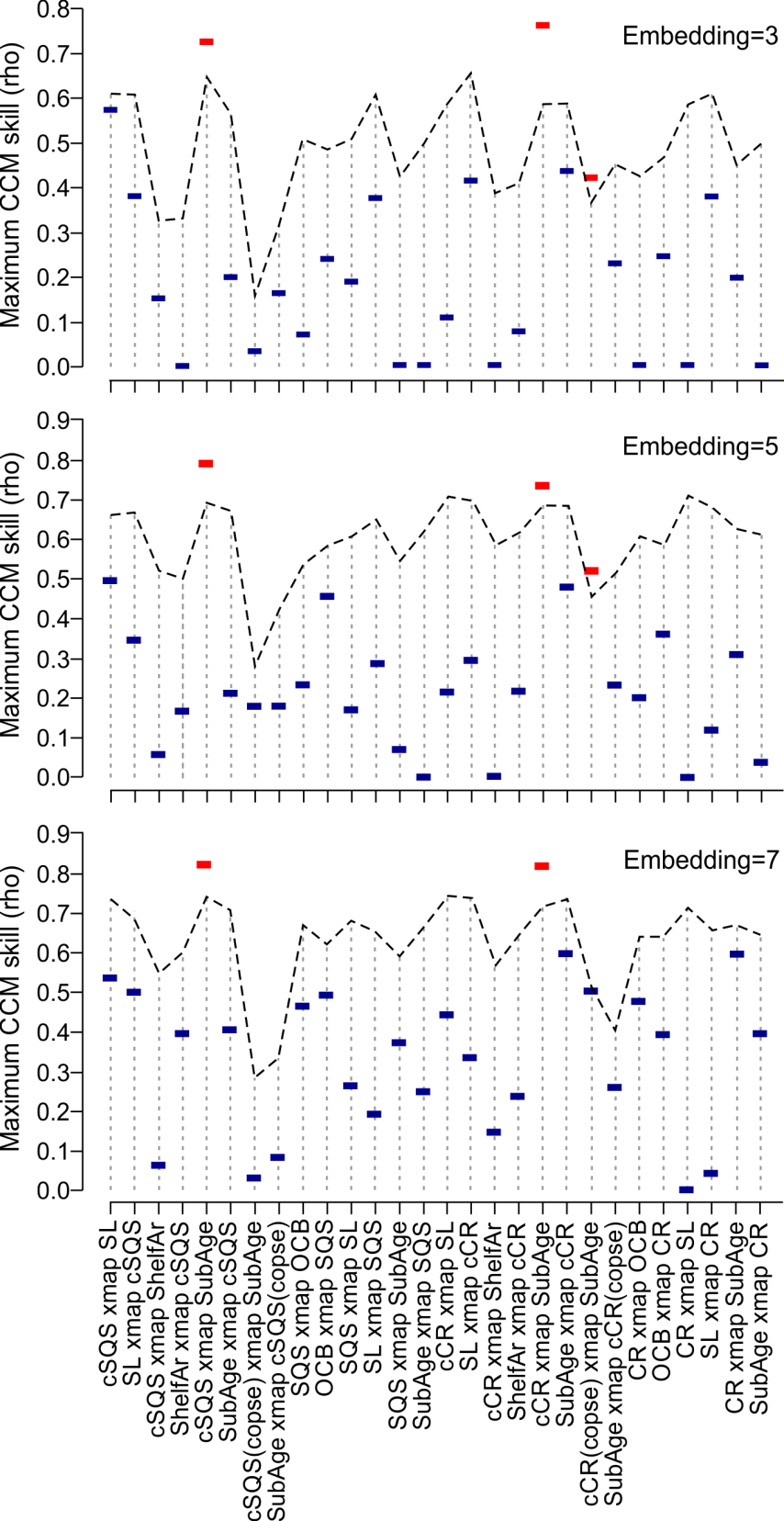
**

**Supplementary Figure S2. Maximum cross mapping skill (blue/red marks) for the whole set of variables investigated.** The corresponding 95^th^ percentiles for 1000 surrogate time series from the Ebisuzaki phase shift null model are also shown (black dashed line). For clarity vertical lines are added to help to identify cross mapped variables listed at the bottom of the plot. Cross-mapping skill and causality was considered significant if the Sugihara’s correlation coefficient (rho) for the cross‑mapping of time series *X* to *Y* exceeded the 95^th^ percentile of the corresponding estimate for the surrogates (e.g., *X* xmap *Y* means that *Y* causes *X*) (denoted by red marks). Significant causal relationships were marked in red. SQS = shareholder quorum subsampling diversity (#genera); CR = classical rarefaction diversity (#genera); OCB = marine organic C burial rate (Tmol C y^-1^); cSQS = SQS diversity corrected for changes in OCB computed from GEOCARBSULF; cCR = as cSQS but for CR diversity estimates; cSQS(copse) = SQS diversity corrected for changes in OCB computed from COPSE model; cCR(copse) = as cSQS(copse) but for CR diversity estimates; SubAge = mean age of subducting crust (Myr); SL = sea level (m); ShelfAr = global shelf area (10^6^ km^-2^).


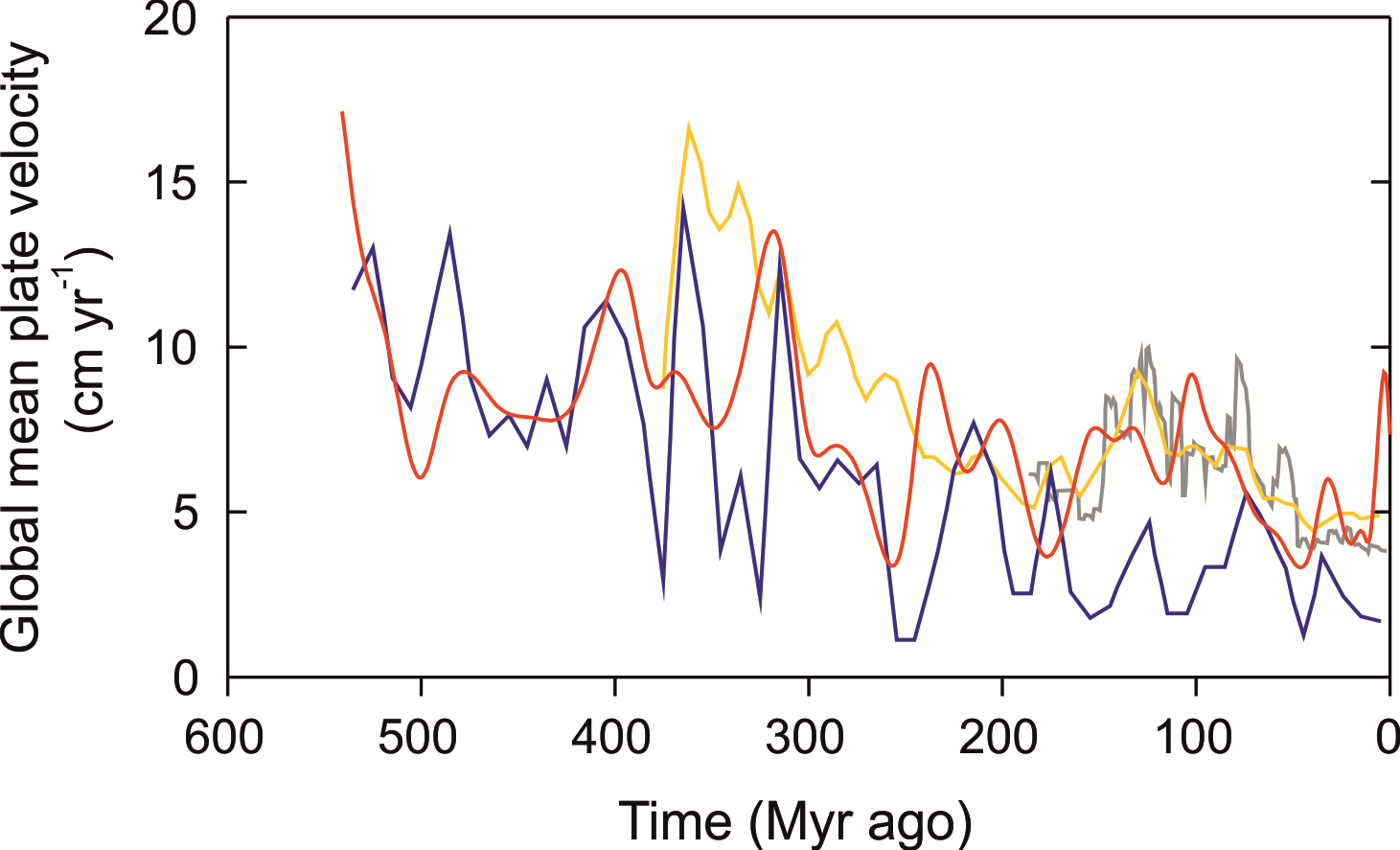


**Supplementary Figure S3. Comparison of average plate velocities computed from different geodynamic models: red, blue**[**^3^**](#_ENREF_3)**, yellow**[**^4^**](#_ENREF_4) **and gray lines**[**^5^**](#_ENREF_5)**.**

**References**

1 Stampfli, G. M. & Borel, G. D. A plate tectonic model for the Paleozoic and Mesozoic constrained by dynamic plate boundaries and restored synthetic oceanic isochrons. *Earth and Planetary Science Letters* **196**, 17-33 (2002).

2 Vérard, C., Hochard, C., Baumgartner, P. O., Stampfli, G. M. & Liu, M. Geodynamic evolution of the Earth over the Phanerozoic: Plate tectonic activity and palaeoclimatic indicators. *Journal of Palaeogeography* **4**, 167-188 (2015).

3 Matthews, K. J. *et al.* Global plate boundary evolution and kinematics since the late Paleozoic. *Global and Planetary Change* **146**, 226-250 (2016).

4 Domeier, M. & Torsvik, T. H. Plate tectonics in the late Paleozoic. *Geoscience Frontiers* **5**, 303-350, doi:http://dx.doi.org/10.1016/j.gsf.2014.01.002 (2014).

5 Zahirovic, S., Müller, R. D., Seton, M. & Flament, N. Tectonic speed limits from plate kinematic reconstructions. *Earth and Planetary Science Letters* **418**, 40-52, doi:https://doi.org/10.1016/j.epsl.2015.02.037 (2015).

**Supplementary Dataset (*.xlsx file)**. Datasets of marine organic C burial rates, marine invertebrate diversity (uncorrected and corrected), sea level height, global shelf area and subducting crustal age used in this study. The original datasets (raw data) are available from the authors upon request.
